# Function and structural regulation of the carbon monoxide (CO)-responsive membrane protein PGRMC1

**DOI:** 10.3164/jcbn.17-132

**Published:** 2018-04-11

**Authors:** Yasuaki Kabe, Hiroshi Handa, Makoto Suematsu

**Affiliations:** 1 Department of Biochemistry, Keio University School of Medicine, 35 Shinanomachi, Shinjuku-ku, Tokyo 160-8582, Japan; 2 Core Research for Evolutional Science and Technology (CREST), Japan Agency for Medical Research and Development (AMED), 20F Yomiuri Shimbun Bldg, 1-7-1 Otemachi, Chiyoda-ku, Tokyo 100-0004, Japan; 3 Department of Nanoparticle Translational Research, Tokyo Medical University, 6-1-1 Shinjuku, Shinjuku-ku, Tokyo 160-8402, Japan

**Keywords:** heme, carbon monoxide, cancer, chemoresistance, metabolism, neuron

## Abstract

Progesterone receptor membrane associated component 1 is a multifunctional heme-binding protein that plays a role in several biological processes such as tumor progression, metabolic regulation, and viability control of nerve cells. Notably, progesterone receptor membrane associated component 1 is highly expressed in various types of cancer cells, and facilitates cancer proliferation and chemoresistance. Recently, progesterone receptor membrane associated component 1 structure has been explored by X-ray crystallographic analysis. Interestingly, whereas apo- progesterone receptor membrane associated component 1 exists as a monomer, the heme-bound progesterone receptor membrane associated component 1 converts into a stable dimer by forming a unique heme-heme stacking structure, leading to activation of epidermal growth factor receptor signaling and chemoresistance in cancer cells. Furthermore, the gas mediator carbon monoxide inhibits progesterone receptor membrane associated component 1-mediated activation in cancer cells by dissociating the heme-stacking dimer of progesterone receptor membrane associated component 1. The dynamic structural regulation of progesterone receptor membrane associated component 1 will provide new insights for understanding the mechanisms underlying its various functions.

## Carbon Monoxide as a Gas Mediator

Gas mediators such as molecular oxygen (O_2_), nitric oxide (NO), carbon monoxide (CO), or hydrogen sulfide (H_2_S) play a role in various physiological functions.^(1)^ It is well known that CO causes chemical hypoxia by inhibiting oxygen transport through binding to the ferrous heme iron of hemoglobin, and stabilize R-state conformation to inhibit O_2_ dissociation. Normally, cytochrome *c* oxidase in the mitochondrial electron transport system is not affected by CO because the heme iron in this enzyme is present as a ferric form under normoxia conditions that does not allow its binding. However, under hypoxia, the heme iron becomes ferrous to allow its binding to block the respiratory chain. Furthermore, exposure to abundant levels of CO causes gas exchange from NO-bound hemeproteins in nervous tissues, resulting in CO-induced, delayed-onset neurotoxicity by facilitating release of NO gas in the brain.^(2,3)^ CO gas is endogenously generated in various organs or cells by the heme-degrading enzyme heme oxygenase. Two isozymes exists; HO-1 and HO-2 as indicible and constitutive forms, respectively.^(1,4–9)^ It has been reported that the endogenously produced CO plays a role in various physiological actions such as vasodilation and contractile action, immune regulation, and cellular defense.^(1)^ However, little has been unveiled about the CO-responsive receptor proteins.

## Identification of PGRMC1 as a CO-Responsive Receptor

**Fig. 1:**
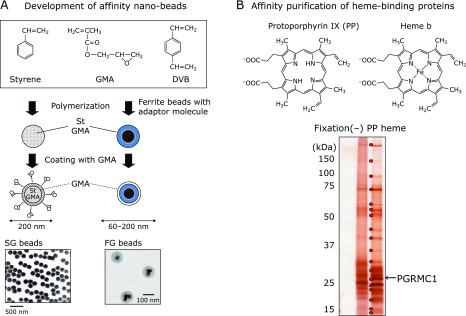
Identification of the CO-responsive protein as PGRMC1 using affinity nano-beads. (A) Development of affinity nano-beads. High performance affinity nano-beads composed of glycidylmethacrylate (GMA)-covered GMA-styrene copolymer core (SG beads) and ferrite particle containing magnetic beads (FG beads). (B) Affinity purification of heme-binding proteins. Using heme- or heme precursor protoporphyrin IX (PP IX)-fixed affinity beads, bound proteins were purified from a mouse liver membrane fraction. PGRMC1 (indicated by an arrow) was identified by ESI-MS.

Because the CO gas is chemically inactive and cannot directly access the target protein, it exerts its function mainly by binding to the prosthetic group heme (iron) on the protein. We have developed high performance affinity nano-beads composed of a glycidylmethacrylate (GMA)-covered GMA-styrene copolymer core (SG beads) and ferrite particle-containing magnetic beads (FG beads),^(10–13)^ as shown in Fig. 1A. Use of theses functionalized beads allowed rapid purification of target proteins for a wide range of chemical compounds with high recovery from protein libraries such as crude cell extracts.^(13)^ We succeeded in isolating and identifying various target proteins for various drugs such as immunosuppressors, antibiotics, and anti-cancer or anti-inflammation agents.^(11,14–16)^ We also identified several novel heme binding factors and the corresponding regulatory mechanisms.^(17,18)^ Based on these properties, we attempted to identify candidate CO-responsive proteins by systematically screening heme iron-specific binding proteins and using heme iron or heme precursor porphyrin protoporphyrin IX (PP) as a control. As a result, several proteins were selectively bound to heme iron from mouse liver extract. Using ESI-MS peptide sequencing, the protein with approximately 25 kDa was identified as the membrane protein PGRMC1. Heme binding and CO response of PGRMC1 was analyzed using ultraviolet absorbance spectra. Soret peaks at 399 and 423 nm were observed for the ferric (Fe III) and ferrous heme state, respectively, of heme-bound PGRMC1, and the peak was further shifted to 419 nm by addition of CO gas. Thus, PGRMC1 is considered a CO gas-responsive candidate protein.

## PGRMC1 Forms a Unique Heme-Stacking Dimer Structure

**Fig. 2:**
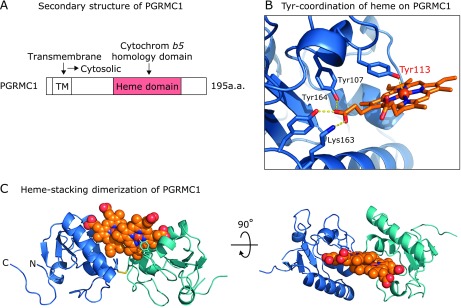
Heme-stacking dimer structure of PGRMC1. (A) Secondary structure of human PGRMC1 protein. TM indicates transmembrane domain. A heme-binding motif is present in the central region of PGRMC1, which shares homology with cytochrome *b*5. (B) Tyr-coordination of heme on PGRMC1. X-ray crystallographic analysis (PDB accession code: 4X8Y) revealed that heme forms a 5-coordinated bond with tyrosine residue 113 of PGRMC1. (C) Heme-stacking dimerization of PGRMC1. Two PGRMC1 subunits (blue and green ribbons) dimerize via stacking of the heme molecules.

PGRMC1 was first identified as a membrane protein with bound progesterone.^(19–21)^ Furthermore, it has been reported that PGRMC1 is highly expressed in various cancer cells^(22,23)^ and is associated with tumor progression and chemoresistance,^(24,25)^ although its structural regulation remains unclear. The structure of PGRMC1 contains a single transmembrane region at the N terminus and a heme binding motif that shows homology to cytochrome *b5* at the central region, which is localized on the cytoplasm (Fig. 2A).^(22)^ Whereas cytochrome *b5* binds to heme through a 6-coordinate bond two axial histidine residues, PGRMC1 lacks these conserved histidine residues. Therefore, it had been unclear how heme is bound to PGRMC1. To elucidate the structural regulation of PGRMC1, we investigated the heme-bound PGRMC1 structure using X-ray crystallographic analysis.^(26)^ The heme iron forms a 5-coordinated bond with tyrosine residue 113 of PGRMC1. Because heme is a hydrophobic prosthetic group, it is generally located inside proteins, although the 5-coordinated heme on PGRMC1 appears unstable because it is exposed on the surface of the protein. In the crystal, we found that PGRMC1 forms a stable dimer through stacking interactions of two protruding heme molecules, without participation of amino acid residues. Such protein dimerization via heme-heme stacking has not been previously seen in eukaryotes, and we termed it “heme-stacking dimer”.

## Cancer Regulation by the Heme-Stacking PGRMC1

The heme group binds to PGRMC1 with of low affinity (*K*_d_ of 50 nM), as determined by heme titration analysis.^(26)^ This binding affinity is comparable to that of iron regulatory protein 2 (IRP2), which is known to be regulated by intracellular levels of heme,^(27)^ suggesting that PGRMC1 forms a heme-stacking dimer in response to the intracellular heme concentration. Furthermore, biochemical analyses such as gel filtration chromatography, native MS, or sedimentation velocity analytical ultracentrifugation (SV-AUC), revealed that apo-PGRMC1 (heme free) exists as a monomer and that it dimerizes by binding with heme. Because PGRMC1 dimerization involves the open surface of heme on the opposite side of the axial Tyr113, no space for CO binding is available in the dimeric structure. Interestingly, CO gas interferes with PGRMC1 dimerization by binding to the 6-coordination site of heme. Thus, the conformation of PGRMC1 is regulated in response to intracellular levels of heme or CO.

As mentioned above, PGRMC1 is highly expressed in various types of cancers. We revealed that the heme-stacking PGRMC1 dimer interacts with the EGF receptor (EGFR), which is involved in signal transduction during cancer proliferation, whereas the apo- or CO-bound PGRMC1 monomer does not bind to the EGFR. In addition, EGFR signalling in colorectal cancer HCT116 cells, auto-phosphorylation of EGFR, and its downstream phosphorylation of AKT or ERK were all inhibited by PGRMC1 knockdown or by the addition of CO. Similarly, reduction of intracellular levels of heme by addition of a heme synthesis inhibitor, succinyl acetone, interferes with EGFR signalling. Furthermore, PGRMC1 knockdown significantly facilitates the anti-cancer effect of the EGFR tyrosine kinase inhibitor erlotinib.

**Fig. 3:**
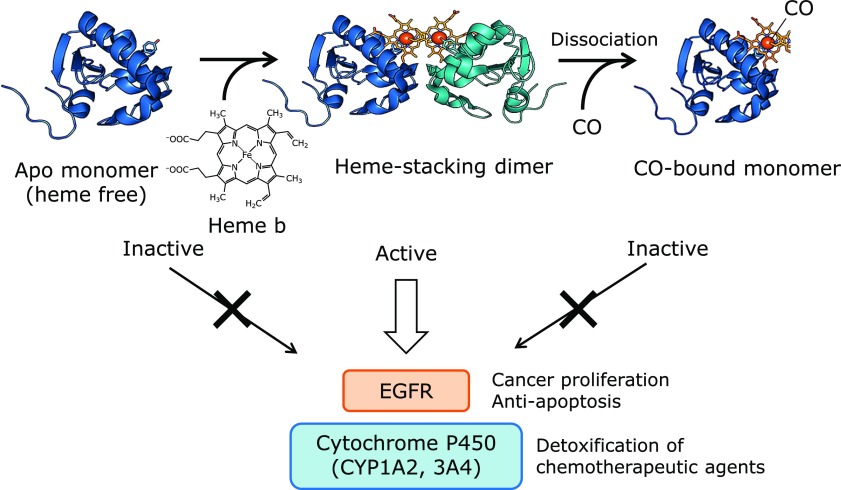
Structural regulation of PGRMC1. Apo-PGRMC1 (heme free) is an inactive monomer. Heme-bound PGRMC1 forms a heme-stacking dimer, enabling the protein to interact with EGFR and cytochrome P450 and leading to activation of cancer proliferation and chemoresistance. CO interferes with the heme-stacking interactions, thereby inhibiting PGRMC1 function.

It has been reported that PGRMC1 interacts with and activates cytochrome P450, including CYP3A4, for drug metabolism^(28)^ or CYP51 for cholesterol synthesis.^(29,30)^ We revealed that the interaction of PGRMC1 with CYP1A2, CYP3A4, or CYP51 is dependent on the formation of the heme stacking dimer. In cancer cells, PGRMC1 facilitates the degradation of the chemotherapeutic agent doxorubicin by activating CYP2D6 or CYP3A4, leading to a reduction of the anti-cancer effect of doxorubicin. Thus, PGRMC1 contributes to cancer proliferation and chemoresistance through interaction with EGFR or cytochrome P450 by forming a unique heme-stacking dimer structure in response to heme concentration in cancer cells. Cancer cells are known to have up-regulated iron uptake,^(31)^ leading to increased heme biosynthesis.^(32)^ It has been reported that PGRMC1 interacts with ferrochelatase (FECH), the terminal enzyme for heme biosynthesis, and regulates its activity by controlling heme release.^(33)^ In addition, a recent report shows that PGRMC1 up-regulates hepcidin expression, which is a peptide hormone produced by hepatocytes that plays a role in iron homeostasis.^(34)^ However, the exact mechanism by which PGRMC1 regulates hepcidin expression remains unclear. PGRMC1 may contribute to the regulation of iron levels in cells through converting into the dimeric structure and binding with heme. On the other hand, as mentioned above, CO gas inhibits the heme stacking dimeric structure of PGRMC1. Excessive heme induces HO-1, the enzyme that oxidatively degrades heme and generates CO. It has been reported that HO-1 induction inhibits the growth of several tumors.^(35,36)^ Therefore, HO-1 induction in cancer cells may inhibit the heme-mediated dimerization of PGRMC1 through the production of CO, and thereby suppress tumor progression. The structural regulation of PGRMC1 is illustrated schematically in Fig. 3. Without heme, PGRMC1 remains an apo-monomer, which has no effect on tumor progression. PGRMC1 appears to interconvert between the apo-monomer and the heme-stacked dimer in response to an increase in heme levels in cells, and stimulates cancer proliferation and chemoresistance by binding to EGFR or cytochrome P450. CO produced by HO-1 induced in response to excess intracellular heme or oxidative stress, inhibits cancer proliferation by dissociating the heme-mediated dimer structure of PGRMC1.

## Regulation and Function of PGRMC1

**Fig. 4:**
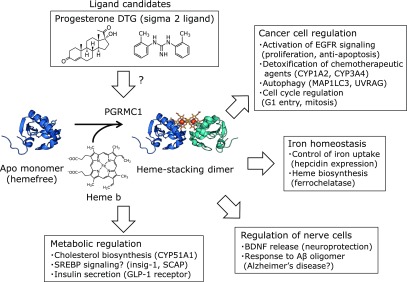
Functions of PGRMC1. Schematic illustration of the various functions of PGRMC1, including regulation of cancer cells, iron homeostasis, metabolism, and nerve cells.

In addition to regulation of EGFR and cytochrome P450, PGRMC1 plays a role in several biological functions (Fig. 4).^(22,23,37)^ In cancer cells, PGRMC1 protects from cell death induced by chemotherapeutic agents such as cisplatin,^(24)^ paclitaxel,^(38)^ and tyrosine kinase inhibitors.^(39)^ Furthermore, PGRMC1 is also associated with resistance induced by several stress responses such as DNA damage^(30,40)^ and oxidative stress.^(41,42)^ Whereas the exact mechanism by which PGRMC1 induces cancer proliferation and chemoresistance has not been fully understood, several regulatory mechanisms have been reported. PGRMC1 and its homolog PGRMC2 are involved in regulating entry into the G1 stage of the cell cycle.^(43)^ In addition, PGRMC1 regulates spindle microtubule stability during mitosis in ovarian cancer cells and interacts with beta-tubulin.^(44)^ PGRMC1 also participates in late events of both mammalian mitosis and oocyte meiosis.^(45)^ Thus, PGRMC1 is thought to activate cell proliferation by regulating several steps during the cell cycle in various cell types. PGRMC1 also participates in autophagy. Mir *et al.*^(46)^ reports that PGRMC1 interacts with the autophagy proteins microtubule-associated protein 1 light chain 3 (MAP1LC3) and UV radiation resistance-associated gene protein (UVRAG), and is required for the lysosomal degradative activity in autophagy.

PGRMC1 participates in the regulation of metabolic pathways. As mentioned above, PGRMC1 is required for cholesterol synthesis by activating CYP51A1, which catalyzes the demethylation of lanosterol.^(29,30)^ Furthermore, PGRMC1 is suggested to be involved in sterol regulatory element-binding protein (SREBP) signaling. Analyses of interactor proteins using photo-crosslinkers revealed that Insig-1 and SREBP cleavage-activating protein (SCAP), components of the SREBP complex in the ER, are binding proteins for PGRMC1.^(47)^ Under cholesterol-starved conditions, SCAP escorts SREBPs to the Golgi complex, where it is activated as a transcription factor and up-regulates the synthesis of enzymes involved in sterol biosynthesis.^(48)^ PGRMC1 is also suggested to regulate membrane trafficking,^(37)^ and might be involved in regulation of SREBP signaling. Besides lipid metabolism, PGRMC1 participates in regulation of insulin secretion. Zhang *et al.*^(49)^ has reported that PGRMC1 interacts with glucagon-like peptide-1 receptor (GLP-1R), and facilitates insulin secretion by increasing the GLP-1-induced cAMP accumulation in pancreatic β cells. Thus, PGRMC1 might participate in obesity or diabetes by regulating these metabolic pathways.

Expression of PGRMC1 (also termed as 25-Dx) is found in various regions of the brain such as choroid plexus, circumventricular organs, hypothalamus, and meninges, and is up-regulated after traumatic brain injury.^(19,50)^ In embryonic sensory neurons, PGRMC1 is suggested to participate in the regulation of Ca^2+^ influx.^(51)^ Another group has reported that release of brain-derived neurotrophic factor (BDNF), a neurotrophin family growth factor that stimulates the survival of neurons, is increased by PGRMC1 via induction of ERK5 signalling.^(52,53)^ Furthermore, recent reports suggest a possible involvement of PGRMC1 in Alzheimer’s disease. Izzo *et al.*^(54,55)^ reports that PGRMC1 contributes to enhanced neurotoxicity by amyloid beta (Aβ) oligomer, and PGRMC1 knockdown lowers Aβ oligomer-binding in neuron cells. Consistent with this, PGRMC1 protects from cell death induced by Aβ oligomer through inhibition of the mitochondrial apoptotic pathway.^(56)^ Thus, PGRMC1 is a potential target for small molecule disease-modifying therapy for Alzheimer’s disease.

PGRMC1 was originally identified as a putative progesterone (P4)-binding membrane component protein by using a ^3^H-labeled-P4 from porcine liver microsomes.^(21)^ Peluso *et al.*^(24)^ reported that the anti-apoptotic effect of P4 is associated with PGRMC1 function in granulosa cells. Another group showed that the effect of BDNF release by P4 mimicked the PGRMC1-mediated pathway.^(52,53)^ In contrast to these results, there is no evidence of direct binding between P4 and PGRMC1. Furthermore, Min *et al.*^(57)^ reported that purified truncated PGRMC1 does not bind to progesterone. Further studies are necessary for elucidating the direct (or indirect) effect of P4 mediated by PGRMC1. Recently, PGRMC1 has been identified as a putative binding protein for sigma-2 ligands,^(58)^ which exhibit an anti-cancer effect and various pathological effects in the nervous system.^(59)^ We have shown that the sigma-2 ligand DTG is able to bind to the heme-stacking dimer of PGRMC1, but the binding affinity is very low (*K_d_* = 84 µM), indicating that PGRMC1 is not the sigma 2 receptor.^(60)^ In addition, TMEM97 was identified as a putative candidate for the sigma 2 receptor with high binding affinity.^(61)^ However, the function of TMEM97 remains unclear. Further analyses will provide insights for the mechanism of action of sigma-2 ligands.

## Conclusion

PGRMC1 is a multifunctional protein that plays a role in several biological processes. PGRMC1 is a potential target for treatment of cancer and several other diseases such as metabolic syndrome or neurological disease. Our results regarding the structure of PGRMC1 clearly showed that the heme-stacking dimerization of PGRMC1 facilitates cancer proliferation and chemoresistance by activating EGFR signaling and detoxification by cytochrome P450. Thus, the development of compounds targeting the PGRMC1 dimer or inhibiting dimerization might be a novel type of therapeutic intervention in cancer. CO gas interferes with the dimerization of PGRMC1 and inhibits cancer proliferation. PGRMC1 also participates in biological processes such as regulation of iron homeostasis and the metabolism of nerve cells. Although the connection between these various functions and the structural regulation of PGRMC1 remains unclear, further analyses should elucidate the unknown mechanism of regulation by CO gas. In conclusion, the dynamic structural regulation of PGRMC1 will provide new insights for an improved understanding of the mechanisms and functions of PGRMC1.
